# Major histocompatibility complex genes exhibit a potential immunological role in mixed *Eimeria*-infected broiler cecum analyzed using RNA sequencing

**DOI:** 10.5713/ab.23.0412

**Published:** 2024-01-20

**Authors:** Minjun Kim, Thisarani Kalhari Ediriweera, Eunjin Cho, Yoonji Chung, Prabuddha Manjula, Myunghwan Yu, John Kariuki Macharia, Seonju Nam, Jun Heon Lee

**Affiliations:** 1 Division of Animal and Dairy Science, Chungnam National University, Daejeon 34134, Korea; 2 Department of Bio-AI Convergence, Chungnam National University, Daejeon 34134, Korea; 3 Department of Animal Science, Uva Wellassa University, Badulla 90000, Sri Lanka

**Keywords:** Chicken, Coccidiosis, Differentially Expressed Gene, Major Histocompatibility Complex (MHC), RNA sequencing

## Abstract

**Objective:**

This study was conducted to investigate the differential expression of the major histocompatibility complex (MHC) gene region in *Eimeria*-infected broiler.

**Methods:**

We profiled gene expression of *Eimeria*-infected and uninfected ceca of broilers sampled at 4, 7, and 21 days post-infection (dpi) using RNA sequencing. Differentially expressed genes (DEGs) between two sample groups were identified at each time point. DEGs located on chicken chromosome 16 were used for further analysis. Kyoto encyclopedia of genes and genomes (KEGG) pathway analysis was conducted for the functional annotation of DEGs.

**Results:**

Fourteen significant (false discovery rate <0.1) DEGs were identified at 4 and 7 dpi and categorized into three groups: MHC-Y class I genes, MHC-B region genes, and non-MHC genes. In *Eimeria*-infected broilers, MHC-Y class I genes were upregulated at 4 dpi but downregulated at 7 dpi. This result implies that MHC-Y class I genes initially activated an immune response, which was then suppressed by *Eimeria*. Of the MHC-B region genes, the *DMB1* gene was upregulated, and TAP-related genes significantly implemented antigen processing for MHC class I at 4 dpi, which was supported by KEGG pathway analysis.

**Conclusion:**

This study is the first to investigate MHC gene responses to coccidia infection in chickens using RNA sequencing. MHC-B and MHC-Y genes showed their immune responses in reaction to Eimeria infection. These findings are valuable for understanding chicken MHC gene function.

## INTRODUCTION

Chicken coccidiosis is a debilitating disease that has imposed a substantial economic burden on the global chicken breeding industry due to declines in productivity, elevated mortality rates, and costs associated with disease prevention [1]. This parasitic disease is caused by protozoa belonging to genus *Eimeria*, which colonize the duodenum, jejunum, ileum, and cecum of chickens, eliciting an inflammatory reaction [1]. Upon *Eimeria* infection, affected chickens exhibit diarrhea, compromised nutrient absorption and growth, and the induction of cellular and humoral immune responses [2]. Enhanced cellular and humoral immune functions play a pivotal role in bolstering resistance against *Eimeria* infection, consequently mitigating intestinal damage and curtailing oocyte development [3,4]. The involvement of T lymphocytes is crucial during coccidiosis infection, as they orchestrate cellular immunity [4]. Therefore, it is essential to improve the immune capacity of chickens to increase resistance to *Eimeria* infection.

The MHC is an important molecular system that profoundly influences the chicken immune response. MHC molecules on cell membranes facilitate crucial cell-to-cell communication within the immune system [5]. Notably, when cells are infected by pathogens, antigen-presenting cells process and present protein fragments derived from these pathogens, and T cells and natural killer cells identify them, thereby triggering immune responses. During parasite infestation, MHC molecules play a critical role by presenting parasite-derived antigens to T cells, thereby contributing significantly to the adaptive immune system [6]. In addition, MHC class I and II affect intestinal parasite resistance via the microbiome [7]. The resistance of chickens to *Eimeria* infection is related to MHC genetics, with resistance and susceptibility differing depending on the MHC gene haplotype. However, the precise involvement of individual genes within the chicken MHC gene region in the response to *Eimeria* infection remains to be fully elucidated [8].

Many genes on chicken chromosome 16 play significant roles in the immune response [8]. This chromosome harbors an MHC-B gene region responsible for classical antigen presentation and an MHC-Y gene region that includes specialized class I and II genes plus C-type lectin-like genes [8]. Although many functional studies have elucidated the roles of MHC-B genes in disease resistance, the precise association between mutations in MHC-Y genes and disease resistance remains elusive [9,10].

To uncover candidate genes and biological pathways underlying the immune response to disease, researchers have investigated changes in gene expression [11–13]. RNA sequencing technology has enabled comprehensive examinations of gene expression across the genome. By identifying the immune responses of livestock to specific diseases, these studies offer valuable insights into the proteins involved in immune function, the corresponding genes that encode them, and the factors that regulate gene expression. Consequently, gene expression data assumes paramount importance in studies focused on understanding the immune response.

Numerous studies have examined chicken coccidiosis to develop antibiotic alternatives [14,15] and effective anti-coccidial vaccines [16,17], and identified candidate genes associated with immune function [11,18]. Previously, our research team identified chronological changes in gene expression patterns throughout the genome in the cecum of broilers affected by coccidiosis, thereby elucidating key responses and pathways [11]. Building upon these past findings, the present study aims to extend our understanding by investigating the gene expression profiles of chromosome 16, encompassing MHC genes, using an updated reference genome.

## MATERIALS AND METHODS

### Animal care

The animal study was reviewed and approved by the Animal Ethics Committee of Chungnam National University (202012A -CNU-164).

### *Eimeria* infection and sample collection

**Figure 1:**
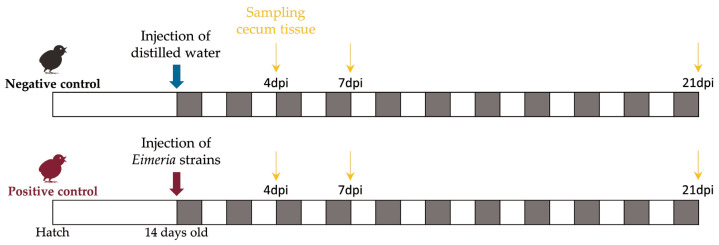
Schematic summary of the experimental design. Modified from Kim et al [11], according to the Creative Commons License. dpi, days post-infection.

Figure 1 summarizes the experimental design. This study used 39 1-day-old male broiler chicks (Indian River; Aviagen, AL, USA). After ensuring that the chicks were free from oocysts, we randomly allocated them to 13 cages. Following a 14-day acclimation period, 21 chickens, referred to as the positive control (PC), were orally administered 1 mL of Livacox T (Biopharm, Prague, Czech Republic), which is a live attenuated vaccine. Each chicken in the PC group received a 10-fold increased dose containing 3 to 5×10^3^ active oocysts of *Eimeria acervulina*, *Eimeria tenella*, and *Eimeria maxima* for infection. The 18 chickens in the non-challenged negative control (NC) were inoculated with distilled water to simulate inoculation stress.

At 4, 7, and 21 days post-infection (dpi), birds in the PC and NC treatments were humanely euthanized. Cecum tissue samples were collected from all chickens and used for RNA extraction. At each time point, three samples from the NC group and five samples from the PC group were randomly selected for RNA sequencing.

### RNA extraction and sequencing

Total RNA was extracted from cecal tissue samples using TRIzol reagent (Invitrogen, Carlsbad, CA, USA). The TruSeq Stranded mRNA sample preparation kit (Illumina, San Diego, CA, USA) was used to construct a cDNA library. Following library construction, all samples were sequenced on the Illumina NovaSeq 6000 platform, which generated 101 bp paired-end reads.

### Sequencing read processing and differentially expressed gene analysis

Adaptor-trimmed sequence data, which we described previously [11], were mapped to the chicken reference genome (GRCg7b, GCA_016699485.1). The reference genome was constructed using bowtie2 v2.5.1 and SAMtools v1.17. STAR v2.7.10b was used for sequence mapping to the reference genome and counting mapped reads. The gene annotation version used was GRCg7b (National Center for Biotechnology Information Annotation Release 106). The *EdgeR* v3.42.4 package in R [19] was used to quantify gene expression. To avoid statistical bias, only genes with a total read count >8 were used for estimating expression levels. Then, the read count was normalized using the trimmed mean of M-value method. Expression levels were compared between the PC and NC groups at every time point. The p-values of the differential expression tests were adjusted using the false discovery rate (FDR) method [20]. Then, genes located on chicken chromosome 16 were selected for the next step. Significant DEGs were defined based on the following criteria: FDR<0.05 and absolute fold change ≥1.5. A lower level of significance for differential expression was also identified within the range 0.05≤FDR<0.1 and absolute fold change ≥1.5. The final DEGs were identified at each time point. Volcano plots depicting the DEGs were generated using the *ggplot2* package in R [21]. To determine the functions of the group of DEGs, gene ontology (GO) and Kyoto encyclopedia of genes and genomes (KEGG) analyses were performed using the Database for Annotation, Visualization, and Integrated Discovery (DAVID). Gene functions were also searched in the relevant literature.

## RESULTS

### Results of RNA sequencing and read mapping

Sequencing of the 24 samples yielded an average of 40,967,230 raw reads per sample after combining forward and reverse sequences. After removing sequencing adapters and trimming low-quality reads, at least 98.29% of the reads remained in all datasets. On average, 40,455,803 reads were retained for subsequent mapping. We aligned the reads to the chicken reference genome, and successfully assembled an average of 37,600,629 reads (approximately 92.95% of the total mapped reads). Table 1 summarizes the sequence data processing.

### Differentially expressed genes between the NC and PC groups

**Figure 2:**
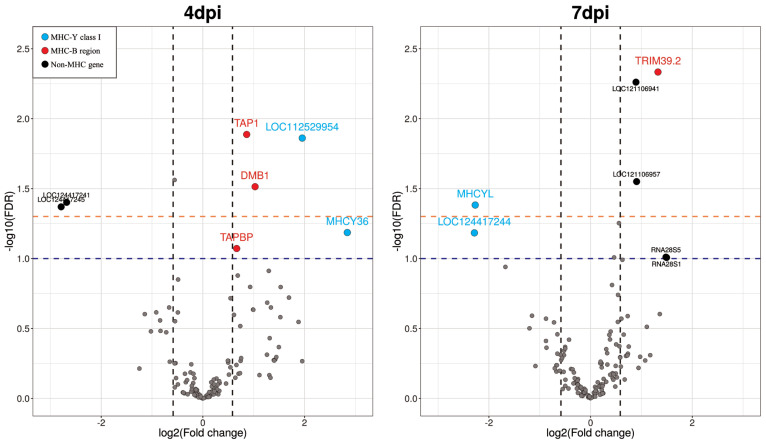
Volcano plots showing significantly differentially expressed genes (DEGs) on chicken chromosome 16 obtained via time-series comparison between positive (PC) and negative control (NC) treatments. The *x*-axis is the log_2_(fold change) and the y-axis the −log_10_(false discovery rate (FDR)) of the tested genes. Vertical dashed lines indicate 1.5-fold changes (up or down). Orange and blue horizontal dashed lines indicate −log_10_ scaled FDR values of 0.05 and 0.1, respectively. Blue dots indicate that the DEGs are MHC-Y class I genes. Red dots are DEGs in the MHC-B region. Black dots are non-MHC genes that belong to neither the MHC-B nor MHC-Y gene region. Because the comparison at 21 days post-infection (dpi) did not show any DEGs, we excluded this time point from the volcano plot.

The chicken chromosome 16 reference genome contained 309 annotated genes. Figure 2 and Table 2 present the findings for 4 dpi, in which five significant DEGs were identified as having FDR<0.05. Of these DEGs, three genes were upregulated in *Eimeria*-infected chickens, whereas two genes were downregulated. Two additional upregulated genes were identified among DEGs with a lower significance threshold (0.05≤FDR<0.1). Upon comparing the NC and PC samples at 7 dpi, we found four significant DEGs having FDR<0.05. Of these genes, three genes were upregulated and only one gene was downregulated in *Eimeria*-infected chickens. Also, there were two additional upregulated genes and one downregulated gene showing a lower significance level (0.05≤FDR <0.1). Notably, no differential expression was observed in any gene at 21 dpi. Therefore, the gene expression comparison results for 21 dpi were excluded from subsequent analyses.

### Categorization of differentially expressed genes based on gene clusters

In *Eimeria*-infected chickens, five genes had significantly higher expression at 4 dpi (FDR<0.1). These genes included *MHCY36* and *LOC112529954*, which are located in the MHC-Y region of the chicken genome, and *DMB1*, *TAP1*, and *TAPBP*, which are located in the MHC-B region. Of the downregulated genes, two (*LOC124417245* and *LOC124417241*) were in the non-MHC region. Notably, at 4 dpi, the most significant DEG was the *TAP1* gene and the largest fold change was for the *MHCY36* gene.

At 7 dpi, five genes were significantly upregulated (FDR<0.1): the *TRIM39.2* gene in the MHC-B region and *LOC121106941*, *LOC121106957*, and two ribosomal RNA genes in the non-MHC region. Two genes (*MHCYL* and *LOC124417244*) downregulated in infected chickens were classified in the MHC-Y class I gene cluster. Among the upregulated genes, the *TRIM39.2* gene had the most significantly upregulated differential expression (FDR = 0.0046). The differential gene expression results are provided in Table 2 and Figure 2.

### Functional analysis of differentially expressed genes

GO analysis conducted on the DEGs at 4 and 7 dpi yielded no significant GO terms. However, KEGG analysis revealed one significant biological pathway (p = 0.03) at 4 dpi, herpes simplex virus 1 infection (gga05168) pathway, which includes the *TAP1* and *TAPBP* genes.

## DISCUSSION

In this study, we examined the roles of chicken MHC genes, which have not been fully characterized functionally. Differential expression of chicken MHC genes was observed in cecal tissues infected with *Eimeria*. This is the first study to focus on MHC gene expression in coccidiosis-infected chickens.

The number of DEGs on chicken chromosome 16 was higher at 4 dpi than at 7 dpi in *Eimeria* infection. This trend of decreasing numbers of DEGs differed from that of a previous study, which observed numerous DEGs across the whole genome as a result of heightened cellular stress and subsequent recovery [11]. These mechanisms were triggered by parasitic attack, rather than being associated with the effects of MHC-related genes. Considering studies that reported a decrease in symptoms after 7 days of coccidial infection [22, 23], we inferred that the immune response of MHC-related genes changed between 4 and 7 dpi. This finding suggests a dynamic immune response during the course of *Eimeria* infection, particularly in relation to MHC genes.

Notably, the pattern of DEGs changed over time following infection. Specifically, the *TAP1* and *TAPBP* genes showed a significant increase in expression at 4 dpi, but not at 7 dpi. *TAP1*, in conjunction with *TAP2*, forms the transporter associated with antigen processing (TAP) complex, which plays a crucial role in processing antigenic peptides for loading onto MHC class I molecules. Additionally, tapasin, which is encoded by the *TAPBP* gene, assists in the interaction between the TAP complex and MHC class I molecules [24]. The action of TAP is essential for processing antigens derived from intracellular protozoan parasites [25]. Therefore, we inferred that antigen presentation via MHC class I molecules was strongly induced at 4 dpi. According to KEGG pathway analyses of DEGs at 4 dpi, TAP and tapasin process antigenic peptides derived from viral proteins and deliver them to MHC class I when a chicken is infected by a pathogen. Our experiment further demonstrated the operation of this pathway.

In comparison, *DMB1* gene expression increased only at 4 dpi. The *DMB1* gene is a non-classical MHC-II gene that plays a role in the transmission of exogenous antigens in the chicken [26]. Parker et al [27,28] reported that the only chicken tissues in which *DMB1* was highly expressed were the intestine and spleen, confirming that *DMB1* is involved in immunity in the infected cecum. As a result, the upregulation of TAP-related genes and the *DMB1* gene at 4 dpi demonstrated the role of both MHC class I and II in *Eimeria* infestation.

Additionally, *MHCY36* and *LOC112529954* were upregulated DEGs at 4 dpi; these genes are functionally related to MHC-Y class I molecules. MHC-Y class I molecules lack the ability to present antigens [8]. However, because MHC-Y genes were significantly upregulated in the cecum at 4 dpi, we infer that MHC-Y class I is responsible for a specific immune response. This finding supports a previous report that MHC-Y genes may be involved in disease resistance [10]. The current result is in line with studies showing the upregulation of chicken MHC-Y genes in response to bacteria and sheep red blood cells [29–31].

Interestingly, the expression pattern of these MHC-Y class I-related genes was reversed at 7 dpi. MHC-Y class I genes were significantly upregulated at 4 dpi, whereas DEGs related to MHC-Y class I genes were downregulated at 7 dpi. According [32], *Eimeria* infection reduces the expression of antimicrobial peptides, and pathogens such as the Marek disease virus induce the downregulation of MHC genes [33], which blocks the host immune response and increases susceptibility to invasion. Thus, *Eimeria* may have suppressed the MHC-Y immune response at 7 dpi.

The *TRIM39.2* gene, which is in the MHC-B region, was differentially expressed at 7 dpi with the highest level of significance (FDR = 0.0046). Although no structural variants or functions of *TRIM* genes have been identified in chickens, based on homology with other species, *TRIM* genes seem to confer innate immunity from viral infection [8,34,35]. There is no evidence that *TRIM39.2* acts in conjunction with MHC-B genes in chickens, and no DEG was found to be functionally related to the classical MHC-B molecule at 7 dpi. As of now, there is no evidence that the *TRIM* genes are functional in intracellular infections in chickens, although they were significantly upregulated in this experiment.

The limited results obtained from the functional analyses using GO and KEGG in this study can be attributed to two factors. First, statistical performance was restricted by the small number of DEGs analyzed. Second, little is known about chicken MHC genes. The number of loci in the MHC-Y gene region was determined only recently [36]; however, the gene functions remain to be studied in many cases. Consequently, we did not anticipate extensive results from the gene function database analysis, as the functions of many recently discovered genes are not known. Future studies should explore the functions of chicken MHC genes, to enable more comprehensive functional analyses.

Importantly, this study elucidated the role of chicken MHC genes under disease conditions. Of particular note is the identification of the association between both MHC-B and MHC-Y genes and the response to pathogen-induced diseases. Although our understanding the precise involvement of the MHC gene in the disease response remains incomplete, further functional genetics research should shed light on this relationship.

## CONCLUSION

We examined the immune response of broiler chickens to *Eimeria* infection at different time points using RNA sequencing and observed the expression patterns of several DEGs on chicken chromosome 16. The results confirmed that MHC classes I and II play roles in immunity during *Eimeria* infection, and that MHC-Y and MHC-B genes were involved in the immune responses. Both gene families changed their differential expression patterns over time. The MHC genes potentially affecting disease resistance reported in this study will be useful for breeding resistant chickens and developing effective drugs and/or vaccines to overcome coccidiosis in the chicken industry.

## Figures and Tables

**Table 1:** Summary of processing sequence data generated from *Eimeria-*infected and uninfected chicken cecum at different time points

Period	Sample ID	Raw reads	Trimmed reads	Trimmed reads ratio (%)	Uniquely mapped read	Uniquely mapped reads ratio (%)
4 dpi	NC4_1	38,168,795	37,724,492	98.84	34,550,630	91.59
	NC4_2	41,692,595	41,135,440	98.66	37,901,703	92.14
	NC4_3	41,677,418	41,127,242	98.68	38,374,220	93.31
	PC4_1	40,485,404	40,015,394	98.84	37,291,833	93.19
	PC4_2	41,718,630	41,100,356	98.52	38,299,348	93.18
	PC4_3	44,287,812	43,727,660	98.74	40,731,044	93.15
	PC4_4	35,459,079	35,051,183	98.85	32,622,878	93.07
	PC4_5	44,735,022	44,182,732	98.77	41,305,106	93.49
7 dpi	NC7_1	44,744,786	44,186,056	98.75	41,365,713	93.62
	NC7_2	44,491,519	43,990,288	98.87	40,793,780	92.73
	NC7_3	42,966,690	42,389,121	98.66	39,686,399	93.62
	PC7_1	35,019,444	34,605,372	98.82	32,246,094	93.18
	PC7_2	43,326,264	42,863,327	98.93	39,947,213	93.20
	PC7_3	44,438,060	43,855,953	98.69	40,825,891	93.09
	PC7_4	39,942,948	39,418,324	98.69	36,674,774	93.04
	PC7_5	40,783,881	40,357,387	98.95	37,150,687	92.05
21 dpi	NC21_1	33,730,093	33,368,276	98.93	31,301,701	93.81
	NC21_2	44,672,291	44,027,266	98.56	40,371,861	91.70
	NC21_3	41,969,337	41,253,407	98.29	38,207,324	92.62
	PC21_1	37,804,068	37,359,671	98.82	34,729,850	92.96
	PC21_2	39,437,362	38,924,098	98.70	36,298,146	93.25
	PC21_3	43,515,518	42,990,643	98.79	39,791,503	92.56
	PC21_4	43,657,011	43,155,250	98.85	40,037,005	92.77
	PC21_5	34,489,491	34,130,326	98.96	31,910,395	93.50
Total		983,213,518	970,939,264	-	902,415,098	-
Average		40,967,230	40,455,803	98.76	37,600,629	92.95

dpi, days post-infection.

**Table 2:** List of differentially expressed genes (DEGs) between the positive (PC) and negative control (NC) treatment groups at 4 and 7 days post-infection with *Eimeria*

Period	Gene symbol	Description	Classification	Regulation	Log_2_(FC)	FDR	Gene type
4 dpi	*LOC112529954*	major histocompatibility complex-Y, class I heavy chain-like	MHC-Y class I	UP	1.9553	0.0138^*^	Protein coding
	*MHCY36*	major histocompatibility complex Y, class I heavy chain 36	MHC-Y class I	UP	2.8424	0.0651	Protein coding
	*TAP1*	transporter 1, ATP-binding cassette, sub-family B (MDR/TAP)	MHC-B region	UP	0.8627	0.0130^*^	Protein coding
	*DMB1*	major histocompatibility complex, class II, DM beta 1	MHC-B region	UP	1.0276	0.0306^*^	Protein coding
	*TAPBP*	TAP binding protein	MHC-B region	UP	0.6659	0.0848	Protein coding
	*LOC124417241*	collagen alpha-1(I) chain-like	Non-MHC region	DOWN	−2.6799	0.0396^*^	Protein coding
	*LOC124417245*	translation initiation factor IF-2-like	Non-MHC region	DOWN	−2.7874	0.0426^*^	Protein coding
7 dpi	*TRIM39.2*	tripartite motif containing 39.2	MHC-B region	UP	1.3251	0.0046^*^	Protein coding
	*LOC121106941*	estradiol 17-beta-dehydrogenase 8-like	Non-MHC region	UP	0.8922	0.0055^*^	Protein coding
	*LOC121106957*	estradiol 17-beta-dehydrogenase 8-like	Non-MHC region	UP	0.9048	0.0282^*^	Protein coding
	*RNA28S5*	RNA, 28S ribosomal 5	Non-MHC region	UP	1.4817	0.0976	rRNA
	*RNA28S1*	RNA, 28S ribosomal 1	Non-MHC region	UP	1.4941	0.0983	rRNA
	*MHCYL*	major histocompatibility complex Y, class I heavy chain like	MHC-Y class I	DOWN	−2.2694	0.0414^*^	Protein coding
	*LOC124417244*	class I histocompatibility antigen, F10 alpha chain-like	MHC-Y class I	DOWN	−2.2863	0.0655	Protein coding

FC, fold change; FDR, false discovery rate; dpi, days post-infection; UP, upregulated differential expression; DOWN, downregulated differential expression.

* FDR<0.05.
